# Wachstumsverläufe der intrinsischen Wertüberzeugungen in Mathematik und Französisch: Zusammenhänge mit Berufsorientierungen

**DOI:** 10.1007/s11618-022-01095-y

**Published:** 2022-05-13

**Authors:** Oana Costache, Peter A. Edelsbrunner, Eva S. Becker, Fabio Sticca, Fritz C. Staub, Thomas Götz

**Affiliations:** 1 Institut für Erziehungswissenschaft, Universität Zürich, Kantonsschulstrasse 3, 8001 Zürich, Schweiz; 2 Institut für Verhaltenswissenschaften, ETH Zürich, Clausiusstrasse 59, 8092 Zürich, Schweiz; 3 Assoziiertes Institut der Universität Zürich, Marie Meierhofer Institut für das Kind, Pfingstweidstrasse 16, 8005 Schweiz Zürich,; 4 Institut für Psychologie der Entwicklung und Bildung, Universität Wien, Universitätsstraße 7, 1010 Wien, Österreich

**Keywords:** Intrinsische Motivation, Berufsorientierungen, Geschlechterunterschiede, Latente Wachstumsklassen, Career aspirations, Gender differences, Intrinsic motivation, Latent growth model

## Abstract

In dieser Längsschnittstudie wurden unterschiedliche Verläufe in der Entwicklung der intrinsischen Wertüberzeugungen in den Unterrichtsfächern Mathematik und Französisch in den Schuljahren 9 bis 11 und deren Zusammenhänge mit Berufsaspirationen untersucht. Bei 850 Schüler*innen von Deutschschweizer Gymnasien (54 % weiblich, Alter T1: 15,6 Jahre) wurden in einem bivariaten Wachstumsmodell fünf Wachstumsklassen identifiziert. Zwei dieser Klassen zeigten eine klare Differenzierung in den Wertüberzeugungen zwischen den beiden Fächern und eine stabile Entwicklung im bevorzugten Fach. Die anderen drei Klassen waren durch Mittelwertunterschiede (hohe, mittlere, niedrige intrinsische Wertüberzeugungen) gekennzeichnet und zeigten moderate Abnahmen über die Zeit in beiden Fächern. Die fünf Wachstumsklassen gingen mit unterschiedlichen Berufsorientierungen am Ende des 11. Schuljahres einher, wobei Berufsorientierungen bezüglich eines Fachs besonders hoch ausgeprägt waren, wenn die Wertüberzeugung bezüglich des anderen Faches niedrig war. Geschlechterunterschiede in den Berufsorientierungen konnten vollständig über die Zugehörigkeit der Geschlechter zu den fünf Wachstumsklassen erklärt werden.

**Zusatzmaterial online:**

Zusätzliche Informationen sind in der Online-Version dieses Artikels (10.1007/s11618-022-01095-y) enthalten.

## Abstract (en)

This longitudinal study investigated different trajectories in the development of intrinsic value beliefs in the subjects Mathematics and French in Grades 9 to 11 and their correlations with career aspirations. Using data from 850 students from German-Swiss high schools (54% female, age T1: 15.6 years), five distinct growth classes were identified in a bivariate growth model. Two of these classes showed clear differentiation between intrinsic value beliefs regarding the two subjects and stable growth in the preferred subject. The other three classes were characterized by mean differences (high, medium, low intrinsic value beliefs) and moderate decline in both subjects. The five growth classes were associated with different career orientations at the end of the 11th grade, with students exhibiting particularly high career orientations in one subject when intrinsic value regarding the other subject was low. Gender differences in career orientations could be fully explained by gender membership in the five growth classes.

## Einleitung

Die subjektiven Wertüberzeugungen von Schüler*innen in Bezug auf Unterrichtsfächer (z. B. Mathematik oder eine Sprache) werden als bedeutsame Prädiktoren für ihr leistungsbezogenes Verhalten und ihre akademischen und beruflichen Entscheidungen postuliert (für Übersichten siehe Eccles 2005; Wigfield et al. 2016, 2017). Jedoch haben verschiedene Längsschnittstudien gezeigt, dass die Wertüberzeugungen über die Schullaufbahn in allen akademischen Domänen abnehmen (z. B. Jacobs et al. 2002; Nagy et al. 2010; Watt 2004). Aufgrund kognitiver Entwicklungsprozesse von Schüler*innen, die bei der Bildung ihrer Wertüberzeugungen mitwirken (Wigfield et al. 2015), wird erwartet, dass sich Schüler*innen auf einige Fachdomänen spezialisieren und sich von anderen abwenden, wenn sie älter werden (Eccles 2009). Beispielsweise könnten sich Schüler*innen entweder auf sprachliche oder mathematische Domänen spezialisieren, was ihre akademische und berufliche Wahl beeinflussen würde (Eccles 2009; Marsh 1986; Möller und Marsh 2013). Zusätzlich gibt es viele Belege für geschlechterspezifische Unterschiede in Wertüberzeugungen und Karriereentscheidungen (für einen Überblick siehe Schoon und Eccles 2014): Schülerinnen bevorzugen und fühlen sich tendenziell kompetenter in sprachlichen Domänen, während Schüler sich in Mathematik (Eccles et al. 1983; Gaspard et al. 2015) und einigen naturwissenschaftlichen Domänen wie Physik und Chemie sehr befähigt fühlen (Jansen et al. 2015).

In Anlehnung an die Erwartungs-Wert-Theorie (EVT, Eccles et al. 1983) und unter Verwendung von Wachstumsanalysen latenter Klassen (Muthén 2004) werden in der vorliegenden Studie Daten aus einer Längsschnittstudie analysiert, um die Differenzierung intrinsischer Wertüberzeugungen in Mathematik und Französisch von Schüler*innen im Verlauf des 9. bis 11. Schuljahres zu untersuchen. Es wird überprüft, ob qualitativ unterschiedliche latente Klassen in den Verläufen der intrinsischen Wertüberzeugungen in Mathematik und Französisch identifiziert werden können (z. B. Schüler*innengruppen, in denen die Wertüberzeugungen in Mathematik sinken, während sie in Französisch relativ stabil bleiben und umgekehrt). Anschließend werden Zusammenhänge zwischen den identifizierten Wachstumsklassen mit dem Geschlecht der Schüler*innen sowie deren Berufsorientierungen bezüglich Mathematik und Französisch untersucht.

## Theoretischer Rahmen

### Wertüberzeugungen und Dimensionale Vergleiche

Erwartungs-Wert-theoretische Ansätze (Eccles et al. 1983) werden oft als Grundlage für die Forschung und die Erklärung von Karriereentscheidungen von Schüler*innen verwendet. Die Grundannahme ist, dass sich Individuen für Aufgaben und Aktivitäten entscheiden, die einen hohen Wert für sie haben und bei denen sie erwarten, dass sie erfolgreich sind. Dabei unterscheiden Eccles et al. (1983) verschiedene Wertkomponenten, die die subjektive Bewertung einer bestimmten Domäne positiv beeinflussen können: Den intrinsischen Wert (Interesse an einer bestimmten Domäne), den Zielerreichungswert (wahrgenommene persönliche Bedeutung einer Domäne) und den Nützlichkeitswert (wahrgenommene Nützlichkeit einer bestimmten Domäne). Die vorliegende Studie fokussiert auf intrinsische Wertüberzeugungen von Schüler*innen in zwei Unterrichtsfächern (Mathematik und Französisch), die unterschiedlichen akademischen Domänen angehören (mathematische und sprachliche Domäne). Es gibt Hinweise darauf, dass intrinsische Wertkomponenten sich bei einigen Schüler*innen bereits zu Beginn der Grundschule ausdifferenzieren (siehe Eccles et al. 1983; Wigfield 1994). Zudem sind intrinsische Wertüberzeugungen ein besonders wichtiger Einflussfaktor für die Bildungs- und Berufswahl von Schüler*innen (Nagy et al. 2006; Watt et al. 2012; Lazarides et al. 2019).

Mehrere Studien haben gezeigt, dass die intrinsischen Wertüberzeugungen von Schüler*innen domänenspezifisch sein können (Gaspard et al. 2017, 2018; Trautwein et al. 2012). Insbesondere gibt es eine starke Unterscheidung zwischen mathematischen und sprachlichen Domänen. Das bedeutet, dass Schüler*innen tendenziell höhere intrinsische Wertüberzeugungen entweder in der mathematischen oder in der sprachlichen Domäne berichten (Möller et al. 2009). Das Bezugsrahmen- oder I/E-Modell von Marsh (1986) kann zu einer Klärung des Phänomens beitragen. Dieses postuliert, dass Wertüberzeugungen durch kontrastierende Vergleiche der Leistungen in den mathematischen und sprachlichen Domänen beeinflusst werden. Tatsächlich haben mehrere Studien gezeigt, dass solche Vergleiche die intrinsischen Wertüberzeugungen von Schüler*innen beeinflussen können (z. B. Gaspard et al. 2018; Guo et al. 2017; Nagy et al. 2008).

### Geschlechterunterschiede in Wertüberzeugungen und Berufsorientierungen

Die wichtige Rolle des Geschlechts für die motivationale Entwicklung von Schüler*innen in den Domänen Mathematik und Sprachen wurde weitgehend belegt (Wigfield et al. 2015). Insgesamt haben Schüler tendenziell höhere intrinsische Wertüberzeugungen in Mathematik, während Schülerinnen tendenziell höhere intrinsische Wertüberzeugungen in den sprachlichen Domänen berichten, obwohl das Ausmaß dieser Geschlechtsunterschiede und ihre Entwicklung im Laufe der Zeit in verschiedenen nationalen Kontexten erheblich variieren kann (Frenzel et al. 2010; Jacobs et al. 2002; Watt 2004). In einer Studie aus den Vereinigten Staaten gaben Schülerinnen beispielsweise an, dass sie Englisch als Unterrichtsfach deutlich höher bewerten als Schüler, was sich nicht nur positiv auf ihre Berufsaspirationen im Bereich Personaldienstleistungen auswirkte, sondern auch negativ auf ihr Interesse an mathematischen und naturwissenschaftlichen Berufen (Lauermann et al. 2015). Ferner berichteten Lazarides und Lauermann (2019) in ihrer Längsschnittuntersuchung in Deutschland, dass Schülerinnen häufiger als Schüler Karrierepläne in sprachlichen Bereichen angaben.

Auch gibt es einige Hinweise, dass stereotype geschlechtsspezifische Unterschiede in den Wertüberzeugungen als Mediatoren zwischen dem Geschlecht der Schüler*innen und ihrer Bildungs- und Berufswahl wirken können. Diese mediierende Funktion wurde empirisch in Längsschnittstudien belegt, so dass indirekte Effekte des Geschlechts auf Berufsaspirationen und Kurswahlen durch die Leistung, das Selbstkonzept und verschiedene Wertkomponenten vermittelt wurden (vgl. Jansen et al. 2021). Andere Studien haben jedoch herausgefunden, dass Geschlechterunterschiede bei mathematikbezogenen Berufsorientierungen bestehen bleiben und nicht (vollständig) durch das mathematikbezogene Selbstkonzept oder die Wertkomponenten mediiert werden (Lauermann et al. 2015, 2017).

### Entwicklung von Wertüberzeugungen und Zusammenhänge mit Berufsorientierungen

Eine große Anzahl von Studien aus verschiedenen Ländern beschreiben eine graduelle Abnahme der intrinsischen Wertüberzeugungen für verschiedene Unterrichtsfächer über die Schulzeit hinweg (siehe Scherrer und Preckel, 2019 für eine Übersicht). Teilweise werden kurvenförmige Trends berichtet, mit stärkerem Rückgang in den ersten Jahren und einer Abflachung in der späten Adoleszenz (Jacobs et al. 2002; Watt 2004). Eine Erklärung dafür ist, dass die Interessen der Schüler*innen mit dem Alter sowohl spezifischer als auch stabiler werden (Krapp 2002; Schiefele 2009). Intraindividuelle Unterschiede der Wertüberzeugungen zwischen verschiedenen Unterrichtsfächern sollten daher mit zunehmendem Alter ausgeprägter werden, wobei einige Wertüberzeugungen hoch bleiben, während andere abnehmen. Es ist daher zu erwarten, dass es unterschiedliche Muster in den Verläufen der Wertüberzeugungen geben kann.

Längsschnittuntersuchungen haben im Allgemeinen bestätigt, dass die Verläufe der Wertüberzeugungen von Schüler*innen unterschiedlichen Mustern folgen und dass diese auch ihre beruflichen Entscheidungen vorhersagen können (Archambault et al. 2010; Musu-Gillette et al. 2015; Lazarides et al. 2020; Wang et al. 2016). Das bedeutet, dass Schüler*innen Berufe in einer Domäne wählen, wenn sie vergleichsweise hohe Wertüberzeugungen für diese Domäne im Vergleich zu anderen Domänen haben (siehe Eccles 2009). Solche negativen domänenübergreifenden Zusammenhänge von Wertüberzeugungen (in Mathematik und in sprachlichen Domänen) mit der Kurswahl, der Studienwahl, und den Berufsorientierungen von Schüler*innen wurden bereits in Querschnittstudien dokumentiert (Lauermann et al. 2015; Nagy et al. 2008; Parker et al. 2012). Dabei stellt sich die Frage, ob auch Unterschiede in der Entwicklung von Wertüberzeugungen in kontrastierenden Domänen langfristige Auswirkungen auf die beruflichen Entscheidungen haben können. Erste Untersuchungen dazu zeigen, dass Schüler*innen, deren Wertüberzeugungen in Mathematik stärker abnehmen als jene im Sprachunterricht (in der untersuchten Stichprobe war dies zumeist Unterricht in der Muttersprache Englisch), eher sprachbezogene Berufe anstrebten als Schüler*innen, die stärkere Abnahme im Sprachunterricht zeigten, und vice versa (Gaspard et al. 2020). Ebenso zeigte sich in einer finnischen Stichprobe, dass diejenigen Schüler*innen höhere Berufsaspirationen bezüglich MINT-Fächern aufwiesen, die sich durch zunehmende Wertüberzeugungen in Mathematik und Naturwissenschaften auszeichneten, wobei unterschiedliche Verlaufsmuster in Wertüberzeugungen Geschlechterunterschiede in Berufsaspirationen teilweise erklären konnten (Guo et al. 2018). Diese Studien legen nahe, dass die Untersuchung von Verläufen über Domänen hinweg vielversprechend ist, um besser zu verstehen, wie intrinsische Wertüberzeugungen mit den Berufsorientierungen der Schüler*innen zusammenhängen.

## Forschungsfragen und Hypothesen

In der vorliegenden Untersuchung werden Verläufe in den intrinsischen Wertüberzeugungen von Deutschschweizer Schüler*innen in Mathematik und Französisch und deren Zusammenhänge mit beruflichen Orientierungen in diesen beiden Domänen untersucht. Der Fokus auf die Domänen Mathematik und Französisch wurde gewählt, weil Französisch und Mathematik im Allgemeinen als sehr schwierige akademische Domänen erlebt werden (z. B. Graham 2004; Haag und Götz 2012), und da es in diesen beiden Domänen anhaltende geschlechtsspezifische Unterschiede bei der Bildungs- und Berufswahl gibt, wobei Schülerinnen wesentlich seltener als Schüler einen Beruf anstreben, der ein hohes Maß an Mathematikkenntnissen erfordert (z. B. Watt et al. 2017). Dabei ist auch zu berücksichtigen, dass der Französischunterricht im Kontext der Schweiz eine spezielle Rolle einnimmt, da es sich bei Französisch um eine der vier offiziellen Amtssprachen der Schweiz handelt. Ziel ist es, die folgenden Forschungsfragen zu beantworten:

1. Können qualitativ unterschiedliche latente Klassen in den Verläufen der intrinsischen Wertüberzeugungen von Schüler*innen in Mathematik und Französisch identifiziert werden? Basierend auf den Studien von Gaspard et al. (2020) und Guo et al. (2018) wird erwartet, dass es Schüler*innengruppen gibt, die in beiden Domänen stabil hohe oder niedrige intrinsische Wertüberschätzung berichten, oder auch Schüler*innengruppen, bei denen die intrinsische Wertüberzeugung in einer Domäne hoch bleibt, in der anderen Domäne aber abnimmt.
2. Gibt es geschlechtsspezifische Unterschiede in der latenten Klassenzugehörigkeit? Auf der Grundlage früherer Untersuchungen über die Zusammenhänge zwischen diesen Variablen und der Entwicklung der domänenspezifischen Wertüberzeugungen (z. B. Gaspard et al. 2020; Lauermann et al. 2015) ist zu erwarten, dass Schüler in Klassen mit positiveren Verläufen (weniger Abnahme) in Mathematik überrepräsentiert sind, während Schülerinnen in Klassen mit positiveren Verläufen in Französisch überrepräsentiert sind.
3. Hängt die Zugehörigkeit zu einer latenten Klasse mit den Berufsorientierungen der Schüler*innen gegen Ende des Gymnasiums zusammen? Zu erwarten ist, dass Schüler*innen in latenten Klassen mit einem positiveren Verlauf (im Durchschnitt sowie über die Zeit) in Mathematik vergleichsweise mit höherer Wahrscheinlichkeit eine positivere Orientierung (höhere Mittelwerte) gegenüber Berufen berichten, die einen mathematischen Fokus haben. Zu erwarten ist auch, dass beispielsweise Schüler*innen mit negativeren Verläufen in Mathematik mit höherer Wahrscheinlichkeit einen Beruf mit einem sprachbezogenen Fokus (Französisch) anstreben.
4. Können Geschlechterunterschiede in der Zugehörigkeit zu einer latenten Klasse Geschlechterunterschiede in den Berufsorientierungen erklären? Auf der Grundlage von früheren Befunden, die eine mediierende Funktion des Geschlechts dokumentierten (z. B. Guo et al. 2018), wird erwartet, dass die Klassenzugehörigkeit Geschlechterunterschiede in den Berufsorientierungen zumindest teilweise erklärt.

## Methode

### Stichprobe und Datenerhebung

Zur Untersuchung der Forschungsfragen wurden Fragebogendaten aus einer Längsschnittstudie mit drei Messzeitpunkten verwendet, die zwischen 2012 (Klasse 9) und 2014 (Klasse 11) in der deutschsprachigen Schweiz an acht Gymnasien durchgeführt wurde. Die Fragebögen wurden in der regulären Unterrichtszeit unter der Aufsicht von geschultem wissenschaftlichem Personal in Papierform bearbeitet. Die Teilnahme war für die Schüler*innen zu jeder Zeit freiwillig. Gleichwohl konnten keine Abbrüche oder Teilnahmeverweigerungen der anwesenden Schüler*innen verzeichnet werden. Nach Ausschluss eines Gymnasiums mit 146 teilnehmenden Schüler*innen, an dem die Schulleitung die Teilnahme nach der ersten Erhebung aus organisatorischen Gründen beendete, bestand die endgültige Stichprobe aus 850 Deutschschweizer Schüler*innen (*M*_Alte r_ = 15,61 Jahre bei T1, *SD* = 0,38; 54 % weiblich) aus sieben Schulen und 37 verschiedenen Schulklassen. Von diesen Schüler*innen waren 753 am ersten Messzeitpunkt anwesend, 659 am zweiten und 667 am dritten. Befunde zu Zusammenhängen fehlender Werte mit Eigenschaften der Schüler*innen werden bei den deskriptiven Statistiken berichtet.

Die Mehrheit der Schüler*innen (91 %) gab an, in der Schweiz geboren zu sein. Die Eltern der Schüler*innen verfügten mit einer Maturitätsquote von 47 % (Väter) bzw. 38 % (Mütter) über ein etwas höheres Bildungsniveau als die Gesamtbevölkerung in der Schweiz (Maturitätsquote ca. 25 %, Bundesamt für Statistik 2020). Die Inhalte und Abläufe der Befragung entsprachen, gemäß Entscheid der Ethikkommission der durchführenden Universität, den ethischen Vorgaben der WMA Deklaration von Helsinki.

### Instrumente

Die intrinsischen Wertüberzeugungen in den zwei Unterrichtsfächern wurden zu jedem der drei Messzeitpunkte (Klasse 9 bis 11) erhoben und die Berufsorientierungen wurden in der 11. Klasse erfragt. Die längsschnittliche Zuordnung der Daten erfolgte anhand numerischer Codes.

#### Wertüberzeugungen

Die intrinsischen Wertüberzeugungen in Mathematik und Französisch (Itembeispiel: „[Mathematik/Französisch] ist mir sehr wichtig, unabhängig von der Note“) wurden an allen drei Messzeitpunkten jeweils durch drei Items und mit einer fünfstufigen Ratingskala von 1 (stimmt gar nicht) bis 5 (stimmt genau) erfasst. Die Skalen wurden aus der Längsschnittstudie PALMA adaptiert (Pekrun et al. 2007). Die interne Konsistenz geschätzt nach Revelle’s Omega betrug in Mathematik an den drei Messzeitpunkten ω_MT1_ = 0,84, ω_MT2_ = 0,85 und ω_MT3_ = 0,88, sowie in Französisch ω_FT1_ = 0,86, ω_FT2_ = 0,88 und ω_FT3_ = 0,95. Für die Analysen wurden Mittelwerte über die drei Items für jedes der zwei Konstrukte an jedem der drei Messzeitpunkte gebildet.

#### Domänenspezifische Berufsorientierungen

Um die domänenspezifischen Berufsorientierungen der Schüler*innen am dritten Messzeitpunkt zu erheben, wurden Items von TIMSS 2011 (Mullis et al. 2012) und PISA 2006 (OECD 2009) adaptiert. Ähnliche Items wurden auch in einer Studie von Schuster und Martiny (2017) verwendet. Die Items wurden in einer Pilotstudie getestet und wiesen angemessene psychometrische Eigenschaften auf. Berufsorientierungen in Mathematik wurden mit sechs Items gemessen und in Französisch mit sieben Items, die auf einer fünfstufigen Ratingskala von 1 (stimmt gar nicht) bis 5 (stimmt genau) bewertet wurden. Davon waren mehrere Items invertiert (z. B. „Ich möchte später möglichst keinen Job machen, in dem man Französisch sprechen muss“; „Ich möchte später möglichst keinen Job machen, in dem man gute Mathematikkompetenzen benötigt“). Für die Analysen wurden die invertierten Items umkodiert und anschließend Mittelwerte für die zwei Skalen gebildet. Eine zweifaktorielle Faktorenanalyse mit zwei zusätzlichen Methodenfaktoren für die invertierten Items bestätigte die Dimensionalität der beiden Konstrukte, χ^2^_56_ = 87,16, *p* < 0,001,* RMSEA* = 0,025, CI90[0,010; 0,037], *CFI* = 0,995, *SRMR* = 0,022. Die geschätzten internen Konsistenzen für Berufsorientierung in den beiden Fächern betrugen ω_M_ = 0,93 und ω_F_ = 0,92.

### Analysestrategie

Die Grundlage für die Beantwortung der vier Forschungsfragen bildete ein bivariates Wachstumsmodell mit latenten Klassen (latent class growth analysis, im Folgenden abgekürzt LCGA; Muthén 2004) unter Verwendung von Mplus 8.4 (Muthén und Muthén 2017). Dieser Ansatz wurde unter anderem gewählt, da er im Vergleich zu vollständigen Random Intercept-Spezifizierungen die Untersuchung intraindividueller Entwicklungsmuster ermöglicht. Dabei wird auch die interindividuelle Varianz berücksichtigt, was für unsere Forschungsfragen von zentraler Bedeutung ist (Lüdtke und Robitzsch 2021; Pekrun 2021; Rohrer und Murayama 2021). Es handelt sich dabei um eine Variante des allgemeineren Wachstums-Mischverteilungsmodells, in der keine Variation der Wachstumsvariablen modelliert wird. Diese Analysemethode ermöglicht es, unterschiedliche Verläufe von Mittelwerten über die Zeit anhand von Wachstumsklassen abzubilden (Forschungsfrage 1: unterschiedliche latente Klassen in den Verläufen der intrinsischen Wertüberzeugungen in Mathematik und Französisch). Dabei entspricht jede Wachstumsklasse einer homogenen Gruppe von Schüler*innen, die sich in ihren Verläufen ähneln und von den Schüler*innen mit anderen Wachstumsverläufen systematisch unterscheiden. Dieser Ansatz verbindet intra- und interindividuelle Modellierung (vgl. Beltz et al. 2016). Dies wird erreicht, indem Wachstumsverläufe geschätzt werden, die auf intraindividueller Veränderung über die Zeit sowie intraindividuellen Mustern in intrinsischen Wertüberzeugungen über die beiden Schulfächer hinweg basieren. Die Häufigkeiten können dann auf inter-individueller Basis mit anderen Variablen in Beziehung gesetzt werden. Entsprechend ermöglicht diese Modellierung auch, Klassen latenter Entwicklungsverläufe unter Kontrolle von Kovariaten (Forschungsfrage 2: Einfluss des Geschlechts auf Entwicklungsverläufe) zu identifizieren und darüber hinaus den Einfluss dieser Entwicklungsverläufe auf mögliche Outcomes (Forschungsfragen 3 und 4: Zusammenhänge von Entwicklungsverläufen mit Berufsorientierungen) zu überprüfen. Um dies zu erreichen, wurde in einer schrittweisen Modellierungsstrategie die Anzahl der Wachstumsklassen erhöht und dann anhand von Fit-Indices (typischerweise der AIC, BIC, und aBIC, Nylund et al. 2007; sowie laut einer aktuellen Simulationsstudie auch z. B. der AIC3, Edelsbrunner et al. 2021, eingereicht) entschieden, wie viele Wachstumsklassen von sich in ihren Entwicklungsverläufen systematisch unterscheidenden Schüler*innen tatsächlich vorliegen (Forschungsfrage 1). Im bivariaten Modellierungsansatz werden dabei für jede resultierende latente Wachstumsklasse ein Intercept (Mittelwert an T3), ein Slope-Mittelwert (summierte Veränderung von T1 bis T3) sowie eine Slope-Ladung an T2 (entspricht dem Anteil der an T2 bereits eingetretenen Veränderung relativ zur Gesamtveränderung von T1 bis T3) geschätzt. Durch Hinzunahme des Geschlechts der Schüler*innen als Kovariate in der Modellschätzung wurde direkt geschätzt, wie die Anzahl der Schüler*innen, die jede der Wachstumsklassen zeigen, sich zwischen den Geschlechtern unterscheidet (Forschungsfrage 2).

Zur Untersuchung der 3. Forschungsfrage (Zusammenhang von Entwicklungsverläufen der Wertüberzeugungen mit Berufsorientierungen), wurden mittels des bias-corrected hypothesis testing-Verfahrens (BCH; McLarnon und O’Neill 2018) Mittelwerte sowie deren Standardfehler auf den beiden Berufsorientierungs-Variablen für jede der resultierenden Wachstumsklassen geschätzt. Damit wurde untersucht, ob und in welchem Ausmaß sich Schüler*innen mit unterschiedlichen Entwicklungsverläufen in den beiden Fächern in ihrer Berufsorientierung bezüglich der beiden Fächer unterscheiden.

Um die 4. Forschungsfrage (Mediation des Geschlechtereffektes auf Berufsorientierungen über Entwicklungsverläufe der Wertüberzeugungen) zu beantworten, wurden basierend auf dem Modell, welches auch für die dritte Forschungsfrage verwendet wurde (Geschlecht als Prädiktor und Berufsorientierungen als Outcomes der latenten Wachstumsklassen) Mediationsparameter nach McLarnon und O’Neill (2018) berechnet. Hierzu wurde geprüft, ob der Zusammenhang des Geschlechts mit der Berufsorientierung in den beiden Unterrichtsfächern durch die Zugehörigkeit zu einer Wachstumsklasse erklärt werden kann (Schätzung indirekter Effekte nach McLarnon und O’Neill, 2018).

Für die Wachstumsmodelle mit latenten Klassen wurde multivariate Kurtosis-robuste full information maximum likelihood-Schätzung verwendet, unter deren Anwendung teilweise fehlende Daten von Schüler*innen miteinbezogen werden können und Modell-Chi-Quadrat- sowie Standardfehler-Schätzungen für multivariate Kurtosis korrigiert werden. Abhängigkeiten von Schüler*innen innerhalb von Schulklassen wurden mittels Huber-White Sandwich-Schätzer Standardfehler korrigiert (vgl. Maas und Hox 2004). Für die Schätzung der Mediationseffekte wurden anhand Kurtosis-robuster Schätzung 95 % Konfidenzintervalle aus bias-korrigiert gebootstrappten Standardfehlerverteilungen mit 10.000 Stichproben verwendet, welche den Sandwich-Schätzer berücksichtigen. Dies ist in bei Mediationsparametern wichtig, da diese multiplizierten Regressionsparametern entsprechen, deren Verteilungen nicht bekannt sind (Shrout und Bolger 2002).

## Ergebnisse

### Messmodelle

Konfirmatorische Faktorenanalysen mit sechs korrelierten latenten Variablen (für die Wertüberzeugungen in Französisch und Mathematik) an den drei Messzeitpunkten und weiteren vier latenten Variablen zur Abbildung von Residualkovarianzen der vier nicht die latente Metrik bestimmenden Indikatoren über die Zeit bestätigten die dimensionale Struktur (in allen Modellen Kurtosis-robuste ML-Schätzung; χ^2^_102_ = 174,19, *p* < 0,001,* RMSEA* = 0,029, CI90[0,022; 0,036], *CFI* = 0,990, *SRMR* = 0,040). Es konnten laut der Kriterien von Chen (2007) ohne sichtbare Verminderung der Modellpassung schwache Messinvarianz und im Vergleich dazu auch starke Messinvarianz über die Fächer und Zeitpunkte sowie über die Geschlechter hinweg angenommen werden (Tab. 1).

**Table 1:** 

	χ^2^	*Df*	*p*	*CFI*	*RMSEA* [90 % CI]	*SRMR*
Fächer & Zeit: Konfigural	174,19	102	< 0,001	0,990	0,029 [0,022; 0,036]	0,040
Fächer & Zeit: Metrisch	228,54	112	< 0,001	0,984	0,035 [0,029; 0,041]	0,051
Fächer & Zeit: Skalar	323,80	122	< 0,001	0,972	0,044 [0,039; 0,050]	0,061
Geschlechter: Konfigural	310,95	204	< 0,001	0,984	0,035 [0,027; 0,042]	0,042
Geschlechter: Metrisch	347,23	224	< 0,001	0,982	0,036 [0,029; 0,043]	0,055
Geschlechter: Skalar	353,16	232	< 0,001	0,982	0,035 [0,028; 0,042]	0,055

Eine zweifaktorielle Faktorenanalyse mit zwei zusätzlichen Methodenfaktoren für die invertierten Items bestätigte die Dimensionalität der beiden Konstrukte, χ^2^_56_ = 87,16, *p* < 0,001,* RMSEA* = 0,025, CI90[0,010; 0,037], *CFI* = 0,995, *SRMR* = 0,022. Die geschätzten internen Konsistenzen für Berufsorientierung in den beiden Fächern betrugen ω_M_ = 0,93 und ω_F_ = 0,92.

### Deskriptive Statistiken

Deskriptive Statistiken sowie manifeste und latente Interkorrelationen der Studienvariablen sind in Tab. 2 ersichtlich. Das Fehlen der Information zu den Berufsorientierungen der Schüler*innen war weder mit deren Geschlecht noch mit intrinsischen Wertüberzeugungen signifikant korreliert. Das Ausmaß fehlender Daten ist aus den Angaben zu den Stichprobengrößen in Tab. 2 ersichtlich. Sämtliche Unterschiede in unstandardisierten Mittelwerten auf den Wertüberzeugungsvariablen (Antwortskala 1–5) zwischen Schüler*innen mit fehlenden oder nicht fehlenden Daten zur Berufsorientierung waren ≤ 0,15. Der MCAR-Test nach Little (1988) ergab ein nicht-signifikantes Ergebnis (χ^2^(125) = 115,0, *p* = 0,739) welches darauf hinwies, dass die fehlenden Daten nicht substantiell von der missing completely at random-Annahme abwichen.

**Table 2:** 

Variable (Skala: 1 bis 5)	1	2	3	4	5	6	7	8	9
1. Männliches Geschlecht	1	0,25**	0,25**	0,22**	−0,31**	−0,36**	−0,27**	0,36**	−0,23**
2. Intrinsischer Wert Mathematik (T1)	0,24**	1	0,70**	0,64**	−0,07	−0,06	−0,11*	0,64*	−0,09**
3. Intrinsischer Wert Mathematik (T2)	0,22**	0,76**	1	0,73**	−0,02	0,01	−0,06*	0,68*	−0,08**
4. Intrinsischer Wert Mathematik (T3)	0,20**	0,69**	0,79**	1	−0,06	−0,04	−0,03	0,77*	−0,10**
5. Intrinsischer Wert Französisch (T1)	−0,30**	−0,04*	0,00	−0,03**	1	0,71**	0,63**	−0,20**	0,58**
6. Intrinsischer Wert Französisch (T2)	−0,37**	−0,07	0,03	−0,03	0,77**	1	0,72**	−0,20**	0,62**
7. Intrinsischer Wert Französisch (T3)	−0,30**	−0,12*	−0,07	−0,03	0,66**	0,78**	1	−0,22**	0,75**
8. Berufsorientierung Mathematik	0,37**	0,63**	0,66**	0,78**	−0,17**	−0,23**	−0,25**	1	−0,20**
9. Berufsorientierung Französisch	−0,24**	−0,13*	−0,09	−0,14**	0,53**	0,60**	0,74**	−0,26**	1
*M*	46 %	3,03	2,89	2,90	2,91	2,85	2,68	2,49	2,28
*SD*	–	1,05	1,06	1,07	1,03	1,02	1,05	0,97	1,14
*N*	850	752	659	667	752	656	660	621	616
α	–	0,85	0,87	0,86	0,87	0,83	0,86	0,95	0,92

*Anmerkungen.* Latente Korrelationen unter der Diagonalen, manifeste Korrelationen über der Diagonalen

* *p* < 0,05, ** *p* < 0,01

### Identifizierung der Latenten Wachstumsklassen

Zur Beantwortung der ersten Forschungsfrage wurde mittels LCGA die Anzahl vorhandener Wachstumsklassen ermittelt. In Tab. 3 sind die Fit-Indices für die Modelle mit einer bis sechs Klassen dargestellt. Die Fit-Indizes wiesen auf das Modell mit sechs latenten Wachstumsklassen hin. Für die Lösung mit sechs Klassen konnte jedoch trotz einer hohen Anzahl randomisierter Startwerte die beste Log-Likelihood nicht repliziert und somit nicht von Modellkonvergenz am globalen Minimum ausgegangen werden. Zusätzlich zeigte die Sechs-Klassen-Lösung keine theoretisch informativen Klassen, welche nicht schon bei der Fünf-Klassen-Lösung auftraten. Deshalb wurde beschlossen, das Modell mit fünf latenten Wachstumsklassen als informative Variante weiterzuverwenden und zu interpretieren.

**Table 3:** 

Anzahl Klassen	Anzahl Parameter	Log-Likelihood	AIC	AIC3	BIC	aBIC	Entropie
1	12	−6072	12168	12180	12225	12187	NA
2	20	−5693	11456	11446	11521	11457	0,76
3	28	−5473	11002	11030	11134	11045	0,77
4	36	−5316	10704	10740	10875	10761	0,76
5	44	−5252	10592	10636	10800	10661	0,73
6	52	−5203	10510	10562	10756	10591	0,74

Entropie = Reliabilität der Klassenzuordnungen einzelner Schüler*innen

*AIC* Akaike Informationskriterium, *AIC3* Akaike Informationskriterium 3; *BIC* Bayesianisches Informationskriterium, *aBIC* Stichprobengrößen-adjustiertes Bayesianisches Informationskriterium

### Beschreibung der Wachstumsklassen

**Figure 1:**
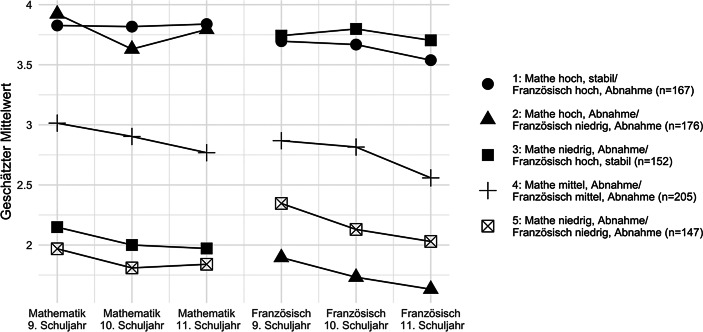

Insgesamt waren die Schüler*innen in etwa zu gleichen Anteilen auf die fünf Wachstumsklassen verteilt (der genaue Anteil als auch die relevanten geschätzten Modellparameter finden sich im Anhang in Tab. A1). Die Wachstumsklassen wurden anhand ihrer Durchschnittswerte sowie ihrer Entwicklung über die Zeit in den beiden Unterrichtsfächern benannt (siehe Abb. 1 und 2). Die erste Wachstumsklasse entspricht Schüler*innen, die in Mathematik stabil über die drei Schuljahre hinweg hohe intrinsische Wertüberzeugungen zeigen, sowie auch in Französisch im 9. Schuljahr hohe, jedoch moderat abnehmende intrinsische Wertüberzeugungen. Diese Wachstumsklasse wurde entsprechend „Mathe hoch, stabil/Französisch hoch, Abnahme“ benannt (19,7 % der Schüler*innen). Schüler*innen dieser Gruppe differenzierten eher wenig zwischen den beiden Unterrichtsfächern. Ein ähnliches Muster zeigte sich für die Wachstumsklassen 4 „Mathe mittel, Abnahme/Französisch mittel, Abnahme“ (24,2 %) und 5 „Mathe niedrig, Abnahme/Französisch niedrig, Abnahme“ (17,4 %), die aber moderate bzw. niedrige intrinsische Wertüberzeugungen für beide Fächer berichteten, welche über die Schuljahre hinweg moderat abnahmen. Ein differenzierendes Muster für die beiden Unterrichtsfächer zeigte sich in der Wachstumsklasse „Mathe hoch, Abnahme/Französisch niedrig, Abnahme“ (20,8 %) und Wachstumsklasse „Mathe niedrig, Abnahme/Französisch hoch, stabil“ (17,9 %). Insgesamt fanden sich somit nur in zwei Wachstumsklassen stabile intrinsische Wertüberzeugungen in jeweils einem der beiden Fächer. Die anderen Verlaufsmuster weisen zumindest geringe und teilweise moderate Abnahme über die drei Schuljahre hinweg auf.

**Figure 2:**
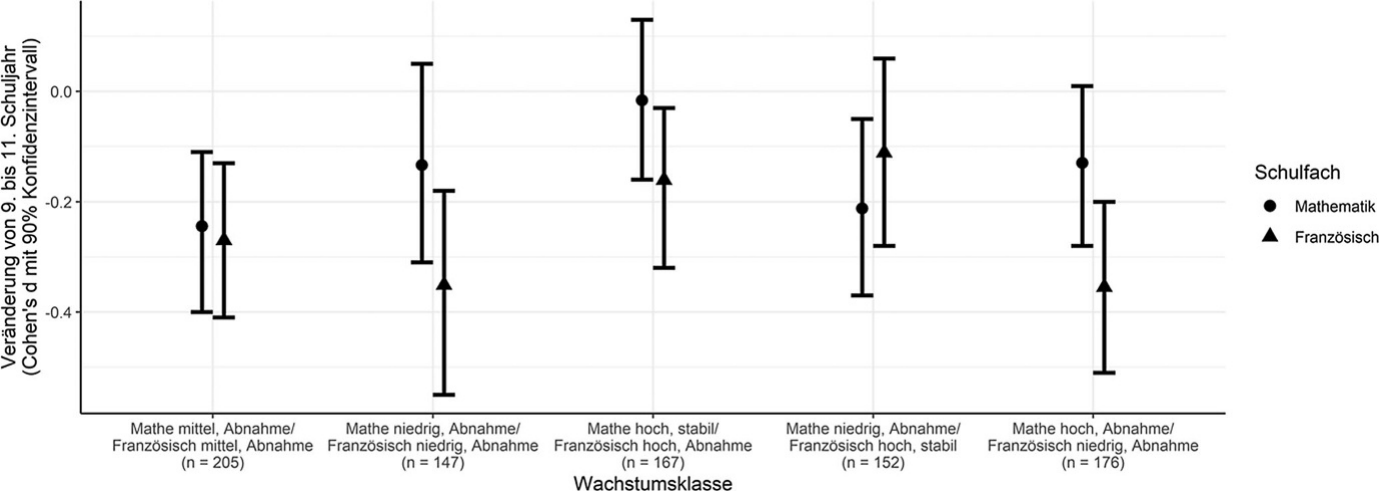

### Geschlechterunterschiede in Entwicklungsverläufen

Um die zweite Forschungsfrage zu beantworten, wird die Verteilung von Schüler*innen auf die fünf Wachstumsklassen betrachtet. Logistische Regressionsparameter aus dem geschätzten Wachstumskurvenmodell bestätigen, dass sich die Geschlechterverteilung auf allen vier Vergleichsklassen mit *p* < 0,05 unterscheidet. Für die fünfte Klasse wird kein Regressionsparameter berechnet, da diese Klasse als die Referenzklasse in der Analyse gilt. Die Unterschiede in der Geschlechtsverteilung innerhalb der Wachstumsklasse 5 können daher nur deskriptiv interpretiert werden. Genauer betrachtet zeigte sich, dass sich in der fächerdifferenzierenden Wachstumsklasse 2 („Mathe hoch, Abnahme/Französisch niedrig, Abnahme“) erwartungsgemäß ein höherer Anteil der Schüler (36 %) befand als jener der Schülerinnen (7 %), während sich in der fächerdifferenzierenden Wachstumsklasse 3 („Mathe niedrig, Abnahme/Französisch hoch, stabil“) ein größerer Anteil der Schülerinnen (26 %) befand als unter den Schülern (9 %). In der Wachstumsklasse 4 („Mathe mittel, Abnahme/Französisch mittel, Abnahme“) befanden sich unter beiden Geschlechtern ein ähnlicher Anteil (23 % der Schülerinnen, 26 % der Schüler), während sich sowohl in der hoch motivierten Wachstumsklasse 1 („Mathe hoch, stabil/Französisch hoch, Abnahme“; 24 % der Schülerinnen, 15 % der Schüler) als auch in der Wachstumsklasse 5 (Mathe niedrig, Abnahme/Französisch niedrig, Abnahme) unter den Schülerinnen mehr (20 %) als unter den Schülern (14 %) befanden.

### Zusammenhänge von Entwicklungsverläufen mit Berufsorientierung

**Figure 3:**
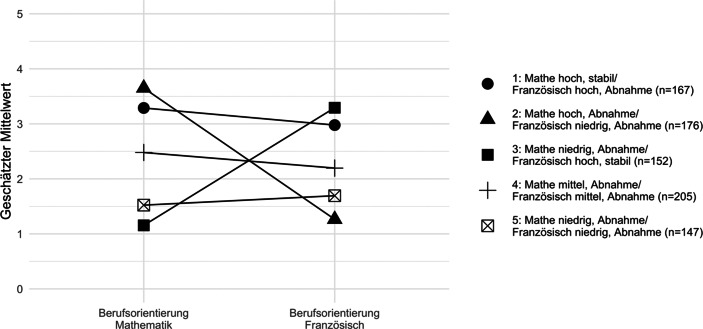

Unter Verwendung des BCH-Verfahrens wurden die Mittelwerte der fünf Wachstumsklassen auf den Berufsorientierungen in Mathematik und Französisch geschätzt, um Unterschiede zwischen den Wachstumsklassen zu untersuchen. Die Ergebnisse der multiplen Gruppenvergleiche zeigten insgesamt signifikante Unterschiede zwischen den Wachstumsklassen im Hinblick auf beide Berufsorientierungs-Variablen (vgl. Tab. A2; Overall-Test, *df* = 4;* Berufsorientierung Französisch global:* χ^2^ = 424,76; *p* < 0,001; *Berufsorientierung Mathematik global:* χ^2^ = 621,98; *p* < 0,001). Dabei zeigte sich, dass Schüler*innen, die das differenzierteste Muster zwischen den Fächern in der Entwicklung ihrer intrinsischen Wertüberzeugen über die beiden Domänen hinweg aufwiesen (Wachstumsklassen 2 und 3), die höchsten Aspirationen für einen Beruf mit Bezug zur entsprechenden Domäne berichteten (vgl. Abb. 3). Schüler*innen, die in beiden Domänen hohe, mittlere oder niedrige intrinsische Wertüberzeugungen aufwiesen, berichteten im Vergleich tiefere Berufsaspirationen in der jeweiligen Domäne. Die Verteilungen der Schüler*innen in den fünf Wachstumsklassen auf den Berufsorientierungen in Französisch und Mathematik sind in Abb. A1 im Anhang veranschaulicht.

### Mediation von Geschlechtereffekten auf Berufsorientierung über Entwicklungsverläufe

**Figure 4:**
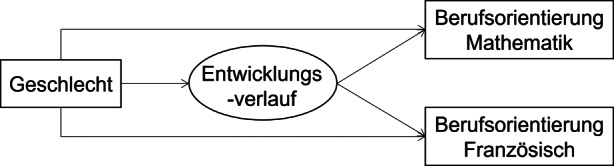

Das Mediationsmodell zur Untersuchung indirekter Effekte des Geschlechts auf die Berufsorientierung über die Wachstumsklassen der Schüler*innen ist in Abb. 4 dargestellt.

Die Ergebnisse aus dem Mediationsmodell sind in Tab. 4 ersichtlich. Als Gesamteffekt wurde für die Berufsorientierung in Mathematik ein Geschlechtereffekt von *b* = 0,83 geschätzt (d. h. Schüler zeigten höhere Mittelwerte in der Berufsorientierung Mathematik im Vergleich zu Schülerinnen), sowie *b* = −0,46 in Französisch (d. h. Schülerinnen berichteten eine höhere Berufsorientierung in Französisch). Dabei zeigte das Modell deutliche Mediationseffekte: In Mathematik konnte der ursprüngliche Geschlechtereffekt vollständig durch einen indirekten Effekt von *b* = 0,75 (Schülerinnen) bzw. *b* = 0,66 (Schüler) über Entwicklungsverläufe der intrinsischen Motivation erklärt werden, und der direkte Effekt reduzierte sich sowohl bei den Schülerinnen als auch bei den Schülern auf geringe Werte deren Konfidenzintervalle 0 einschlossen. In Französisch waren die indirekten Effekte ähnlich groß, mit *b* = 0,65 (Schülerinnen) bzw. *b* = −0,56 (Schüler; negativ, aufgrund des entgegengesetzten Geschlechterunterschieds), wobei der direkte Effekt damit wiederum so stark reduziert wurde, dass er bei beiden Geschlechtern 0 einschloss (vollständige Mediation). In beiden Schulfächern kann die Zugehörigkeit zu einer Wachstumsklasse die Geschlechterunterschiede in der Berufsorientierung entsprechend vollständig erklären.

**Table 4:** 

Effekt des Geschlechts auf Berufsorientierung	Unstd. Effekt	Bootstrap-Konfidenzintervall (95 %)
Indirekter Effekt in Mathematik: Schülerinnen	0,75	[0,48; 1,08]
Indirekter Effekt in Mathematik: Schüler	0,66	[0,41; 1,07]
Indirekter Effekt in Französisch: Schülerinnen	−0,37	[−0,53; −0,21]
Indirekter Effekt in Französisch: Schüler	−0,37	[−0,53; −0,21]
Direkter Effekt in Mathematik: Schülerinnen	0,17	[−0,14; 0,37]
Direkter Effekt in Mathematik: Schüler	0,07	[−0,26; 0,40]
Direkter Effekt in Französisch: Schülerinnen	0,10	[−0,07; 0,49]
Direkter Effekt in Französisch: Schüler	0,19	[−0,12; 0,43]
Gesamteffekt in Mathematik	0,83	[0,57; 1,02]
Gesamteffekt in Französisch	−0,46	[−0,62; −0,32]

Anmerkungen. Da es sich bei dem Geschlecht der Schüler*innen um eine dichotome unabhängige Variable handelt, werden direkte und indirekte Effekte für die beiden Gruppen jeweils einzeln geschätzt, wobei sich der Gesamteffekt jeweils aus dem indirekten Effekt in einer Gruppe und dem direkten Effekt in der anderen Gruppe zusammensetzt (siehe McLarnon und O’Neill 2018)

## Diskussion

In Erweiterung zu bestehenden Studien fokussierte die vorliegende Untersuchung darauf, einen Einblick in die differenzielle Entwicklung von intrinsischen Wertüberzeugungen in Französisch und Mathematik von Schüler*innen am Gymnasium zu geben, sowie Zusammenhänge mit den Berufsorientierungen zu bestimmen. In verschiedenen theoretischen Traditionen zur Motivationsentwicklung wird eine zunehmende Ausdifferenzierung der intrinsischen Wertüberzeugungen und Interessen der Schüler*innen angenommen (Eccles 2009; Schiefele 2009). Dieses Muster der intraindividuellen Differenzierung über Domänen hinweg ist im Wesentlichen mit unseren Ergebnissen vereinbar. Jedoch zeigen wir zusätzlich, dass es auch einen nicht unbeträchtlichen Teil von Schüler*innen gibt, die von diesem Muster abweichen. Die Analyse unserer längsschnittlichen Daten legt dar, dass sich die Verläufe von intrinsischen Wertüberzeugungen in fünf qualitativ unterschiedliche Wachstumsklassen kategorisieren lassen. Es zeigte sich, dass nur zwei Wachstumsklassen eine Differenzierung nach Domänen aufwiesen und die intrinsischen Werte dabei nur in der bevorzugten Domäne über die Zeit hinweg stabil blieben. Im Gegensatz dazu waren die anderen drei Klassen durch Niveauunterschiede (hoch, mittel, tief) und eine moderate Abnahme in Wertüberzeugungen in beiden Domänen gekennzeichnet.

Insgesamt deuten unsere Ergebnisse darauf hin, dass zwar fächerspezifische Entwicklungsverläufe vorliegen, die intrinsischen Wertüberzeugungen der Schüler*innen jedoch dann über die Schuljahre hinweg stabil bleiben, wenn sie differenziert und hoch ausgeprägt sind. Diese Befunde unterscheiden sich teilweise von den Ergebnissen von Gaspard et al. (2020) und Guo et al. (2018), die nur drei Klassen in domänenübergreifenden Verläufen fanden; davon zeigte eine ihrer Klassen einen stabilen Entwicklungsverlauf über *alle* Domänen hinweg. Eine Erklärung dafür, weshalb wir Stabilität nur dann identifizierten, wenn die intrinsischen Werte in einer der beiden Domänen hoch waren, könnte darin bestehen, dass unsere Untersuchung den Vergleich zwischen Mathematik und *Französisch* näher betrachtete. Französisch gilt in der deutschsprachigen Schweiz als Fremdsprache, was möglicherweise die intraindividuellen Domänenvergleiche akzentuiert hat. Die anderen beiden Studien haben hingegen Mathematik/Naturwissenschaften mit den Muttersprachen (English bzw. Finnisch) der Schüler*innen verglichen, weshalb es denkbar wäre, dass die Wertung dieser Domänen über die Zeit stabiler bleibt.

Ferner zeigten sich Geschlechtsunterschiede in der Klassenzugehörigkeit, die mit Geschlechterstereotypen sowie früheren Forschungen im Einklang stehen (z. B. Gaspard et al. 2020; Guo et al. 2018). So waren Schüler in der fächerdifferenzierenden Wachstumsklasse 2 „Mathe hoch, Abnahme/Französisch niedrig, Abnahme“ deutlich überrepräsentiert, während sich in der Wachstumsklasse 3 „Mathe niedrig, Abnahme/Französisch hoch, stabil“ ein größerer Anteil der Schülerinnen befand. Eine Überrepräsentation von Schülerinnen gab es auch in der hoch motivierten Wachstumsklasse 1 „Mathe hoch stabil/Französisch hoch Abnahme“, sowie in der niedriger motivierten Wachstumsklasse 5 „Mathe niedrig, Abnahme/Französisch niedrig, Abnahme“. Dies ist teilweise entgegen bisheriger Forschung und unserer Erwartung, dass Schülerinnen überwiegend positivere Verläufe in den Sprachfächern (Französisch) aufweisen würden, und deutet darauf hin, dass es eine hohe Variation in den intrinsischen Wertüberzeugungen von Schülerinnen in beiden Domänen gibt, die näher betrachtet werden sollte, um mögliche Determinanten solcher Differenzen besser beschreiben zu können.

Die vorliegende Studie erweitert die EVT-basierte Forschung, indem aufgezeigt wird, dass die intrinsischen Wertüberzeugungen der Schüler*innen mit ihren Berufsaspirationen am Ende der Gymnasialbildung zusammenhängen. Die differenzierte Analyse der Berufsorientierungen in Französisch und Mathematik legt dar, dass sich die Aspirationen der Schüler*innen, einen Beruf zu ergreifen, der Französisch- oder Mathematikkenntnisse erfordert, auf die Unterschiede in der Entwicklung der intrinsischen Wertüberzeugungen zurückführen lassen. Die Bedeutung von Wertüberzeugungen für die Berufsaspirationen ist jedoch ausgeprägter, wenn die Schüler*innen eine klare Differenzierung zwischen den zwei Domänen aufweisen. Beispielsweise zeigen unsere Analysen, dass Schüler*innen der Wachstumsklasse 1 mit hohen Wertüberzeugungen in beiden Domänen („Mathe hoch, stabil/Französisch hoch, Abnahme“) ähnlich hohe intrinsische Wertüberzeugungen in Mathematik haben, wie die Schüler*innen der Wachstumsklasse 2 „Mathe hoch, Abnahme/Französisch niedrig, Abnahme“. Trotzdem berichten Schüler*innen der allgemein motivierten Wachstumsklasse 1 schon deutlich niedrigere Berufsorientierungen bezüglich Mathematik (*M* = 3,29) als Schüler*innen der Wachstumsklasse 2 (*M* = 3,65). Dies unterstützt die These von Eccles (2009), wonach intrinsische Wertüberzeugungen nicht nur direkte Prädiktoren sind, sondern auch intraindividuelle Hierarchien in den Wertüberzeugungen die Karriereentscheidungen von Schüler*innen beeinflussen können.

Schließlich untersuchten wir, inwiefern geschlechtsspezifische Unterschiede in der Entwicklung von intrinsischen Wertüberzeugungen Geschlechterunterschiede in den Berufsorientierungen mediieren. Die Mediationsanalyse zeigte, dass der Einfluss des Geschlechts auf Berufsorientierungen in beiden Fächern nach dem Einschluss der Entwicklungsverläufe nicht mehr signifikant von 0 unterschiedlich war und dass in beiden Fächern indirekte Effekte vorlagen. Diese Ergebnisse deuten darauf hin, dass geschlechtsspezifische Verläufe der intrinsischen Wertüberzeugungen die Grundlage dafür mitprägen, ob Schüler*innen später einen Beruf anstreben, der gute Mathematik’ oder Französischkenntnisse verlangt. In praktischer Hinsicht ergibt sich also hier die Möglichkeit für eine frühe Intervention in den beiden Fächern, um die intrinsischen Wertüberzeugungen der Schüler*innen zu fördern und eine mögliche Stereotypenbildung einzudämmen. Aufgrund unserer Befunde und früherer Forschung (Guo et al. 2018) ist es ferner plausibel anzunehmen, dass geschlechtsspezifische Unterschiede in der Entwicklung der Wertüberzeugung die Wahrscheinlichkeit der Wahl eines verwandten Berufes erklären können. Unsere Ergebnisse der vollständigen Mediationen deuten zugleich darauf hin, dass mit den Entwicklungsverläufen potenzielle korrelierte und unbeobachtete Faktoren welche Berufsorientierungen beeinflussen könnten, wie beispielsweise Geschlechtsspezifische Stereotype (Makarova et al. 2019), abgedeckt werden. Die vorliegenden Ergebnisse erlauben beispielswese, Hypothesen über mögliche Interventionsstudien zu formulieren. Es wäre plausibel anzunehmen, dass Maßnahmen, die sich ausschließlich auf die intrinsischen Wertüberzeugungen von Schüler*innen konzentrieren, möglicherweise nicht ausreichen würden, um die Beteiligung von Frauen (oder Männern) in mathematikintensiven Bereichen zu erhöhen.

## Limitationen und Ausblick

Diese Studie weist mehrere Einschränkungen auf, die in der zukünftigen Forschung berücksichtigt werden sollten. Bei der Interpretation der Ergebnisse ist zu beachten, dass Wachstumsklassen, die bei der personenorientierten Analyse identifiziert wurden, explorativer Natur sind. Die vorliegende Stichprobe ist zwar als relativ groß einzustufen, sie bestand jedoch nur aus Schüler*innen aus der deutschsprachigen Schweiz. In Bezug auf die Wahrnehmung von Unterrichtsfächern und die Berufswahlorientierung können soziokulturelle Aspekte und Sozialisationsprozesse eine Rolle spielen, die sich zwischen Ländern und Kulturen unterscheiden (siehe Eccles und Wang 2016). Zukünftige Forschung sollte daher untersuchen, ob ähnliche Wachstumsklassen und Zusammenhänge in verschiedenen Ländern und Schulkontexten gefunden werden können. Dies könnte beispielsweise erreicht werden, indem anhand von Interpretationsregeln, die basierend auf Theorie und vorherigen empirischen Befunden vorab erstellt werden überprüft wird, ob sich über verschiedene Datensätze hinweg Wachstumsklassen zeigen, welche jeweils denselben Interpretationsmustern zuzuordnen sind (vgl. Schiefer et al. 2022). Die Daten der vorliegenden Studie entsprechen zudem nicht-experimentellen Beobachtungsdaten. Längsschnittliche Datenanalysen können auch bei nicht-experimentellen Daten zu kausaler Inferenz beitragen (vanderWeele 2021). Dennoch sollten besonders die Schlussfolgerungen bezüglich der Mediationseffekte mit Vorsicht betrachtet und in zukünftigen Studien weiter abgesichert werden.

Um besser zu verstehen, welche Prozesse die Differenzierung der intrinsischen Wertüberzeugungen der Schüler*innen vorantreiben, ist es ferner notwendig auch weitere als die hier einbezogenen Faktoren zu berücksichtigen. Solche Prozesse könnten beispielsweise leistungsbezogene Einstellungen beinhalten (Möller und Marsh 2013; Schiefele 2009). Ein Ansatz wäre, deren reziproke Effekte mit intrinsischen Werten über die Zeit zu untersuchen.

Eine weitere Einschränkung ergibt sich aus der hier angewandten latenten Klassen-Wachstumsmodellierung, welche keine Varianz der Schüler*innen um die geschätzten Werte ihrer Wachstumskurven erlaubt. Dies wurde versucht zu implementieren, jedoch war die Wachstums-Mischverteilungsmodellierung im bivariaten Ansatz nicht schätzbar. Dies konnte auch durch Restriktionen wie lineare Parametrisierung der Wachstumsverläufe nicht erreicht werden. Solche Schätzproblem sind bei dieser Modellklasse bekannt (McNeish und Harring, 2019) und selbst vier von McNeish und Harring (2019) vorgeschlagene Implementierungen von Residualkovarianzstrukturen konnten als Kompromiss beider Ansätze nicht geschätzt werden. Jedoch ging für unsere Forschungsfragen dadurch keine substantielle Information verloren, da individuelle Wachstumskurven bei uns nicht von Interesse waren.

Trotz dieser Einschränkungen bieten die Befunde eine wichtige Ergänzung zur bestehenden Literatur zur Entwicklung der intrinsischen Wertüberzeugungen von Schüler*innen, indem sie zeigen, dass es qualitativ unterschiedliche Verläufe gibt, die zwischen den Geschlechtern variieren und die Berufsorientierungen von Schüler*innen vorhersagen können. Der von uns gewählte Ansatz entspricht dabei der Verknüpfung intra- und individueller Ansätze (z. B. Beltz et al. 2016). Dies hat den Vorteil, dass die intra-individuell identifizierten Verlaufsmuster über die Zeit sowie über die beiden Schulfächer hinweg stärker auf die dabei beschriebenen Individuen übertragbar sind, was bei Studien die rein inter-individuell basierte Ansätze verfolgen nicht gegeben sein muss (Moeller 2021). Damit bringt unsere Studie Hypothesen für verlaufsspezifische Interventionsstrategien in einer kritischen Phase der Adoleszenz auf, in der sich aufkeimende Zukunftspläne zu konkretisieren beginnen und zeigt, wie wichtig es ist, die Motivationsentwicklung von Schüler*innen intraindividuell (vgl. Dietrich et al. 2019; Molenaar 2004; Murayama et al. 2017) über mehrere Domänen hinweg gemeinsam zu untersuchen.

## Supplementary Information



