# Supplementary material for: Growth trajectories of intrinsic value beliefs in mathematics and French: Relations with career orientations
Source: Z Erziehwiss. 2022 May 13;25(2):269–91. [Article in German] doi: 10.1007/s11618-022-01095-y (PMC9296413; doi:10.1007/s11618-022-01095-y)

**Anhang**

**Tab. A1** Anteile und Wachstumsparameter der fünf identifizierten Wachstumsklassen

| Latente Klasse | Anteil in n (%) | Mathematik | | | Französisch | | |
| --- | --- | --- | --- | --- | --- | --- | --- |
|  |  | Intercept (SE) | Slope (SE) | Slope-Ladung T2 (SE) | Intercept (SE) | Slope (SE) | Slope-Ladung T2 (SE) |
| 1 | 167 (19.7 %) | 3.84 (0.10) | -0.01 (0.07) | 1.84 (9.39) | 3.54 (0.10) | 0.16 (0.08) | 0.82 (0.38) |
| 2 | 176 (20.8 %) | 3.79 (0.10) | 0.13 (0.10) | -1.27 (1.38) | 1.63 (0.07) | 0.26 (0.07) | 0.38 (0.22) |
| 3 | 152 (17.9 %) | 1.97 (0.10) | 0.18 (0.09) | 0.16 (0.36) | 3.70 (0.15) | 0.04 (0.09) | 2.43 (4.47) |
| 4 | 205 (24.2 %) | 2.77 (0.17) | 0.25 (0.10) | 0.55 (0.26) | 2.56 (0.18) | 0.31 (0.12) | 0.83 (0.24) |
| 5 | 147 (17.4 %) | 1.84 (0.13) | 0.13 (0.08) | -0.24 (0.71) | 2.03 (0.12) | 0.32 (0.11) | 0.32 (0.32) |

*Anmerkungen.* *Anteil* beschreibt den geschätzten Anteil an Schüler*innen in jeder der Wachstumsklassen, *Intercept* beschreibt den geschätzten Mittelwert jeder Wachstumsklasse an T3 (11. Schuljahr), *Slope* die Gesamtveränderung von T1 (9. Schuljahr) bis T3 (11. Schuljahr) und Slope-Ladung T2 den geschätzten Anteil der Gesamtveränderung, der im 10. Schuljahr bereits eingetreten ist.

**Tab. A2** Unterschiede in Berufsorientierungen in Französisch und Mathematik angegeben durch Mittelwerte zwischen den Wachstumsklassen

|  | Berufsorientierung Französisch | | |
| --- | --- | --- | --- |
|  | M (*SE*) | | |
| Wachstumsklasse 1 | 2.99 (0.07) | | |
| Wachstumsklasse 2 | 1.27 (0.08) | | |
| Wachstumsklasse 3 | 3.29 (0.09) | | |
| Wachstumsklasse 4 | 2.20 (0.10) | | |
| Wachstumsklasse 5 | 1.69 (0.09) | | |
|  | χ2(*p*-Wert) | | χ2(*p*-Wert) |
| Overall-Test | 424.76 (< .001) |  |  |
| Wachstumsklasse 1 vs. 2 | 38.79 (< .001) | Wachstumsklasse 1 vs. 2 | 11.99 (.001) |
| Wachstumsklasse 1 vs. 3 | 49.05 (< .001) | Wachstumsklasse 1 vs. 4 | 60.95 (< .001) |
| Wachstumsklasse 2 vs. 4 | 145.61 (< .001) | Wachstumsklasse 2 vs. 3 | 133.90 (< .001) |
| Wachstumsklasse 3 vs. 5 | 7.61 (.006) | Wachstumsklasse 2 vs. 5 | 12.51 (< .001) |
| Wachstumsklasse 4 vs. 5 | 276.40 (< .001) | Wachstumsklasse 3 vs. 5 | 262.95 (< .001) |
|  | Berufsorientierung Mathematik | | |
|  | M (*SE*) |  |  |
| Wachstumsklasse 1 | 3.29 (0.09) |  |  |
| Wachstumsklasse 2 | 3.65 (0.08) |  |  |
| Wachstumsklasse 3 | 1.16 (0.09) |  |  |
| Wachstumsklasse 4 | 2.48 (0.10) |  |  |
| Wachstumsklasse 5 | 1.52 (0.10) |  |  |
|  | χ2(*p*-Wert) | | χ2(*p*-Wert) |
| Overall-Test | 621.98 (< .001) |  |  |
| Wachstumsklasse 1 vs. 2 | 32.14 (< .001) | Wachstumsklasse 1 vs. 2 | 39.82 (.001) |
| Wachstumsklasse 1 vs. 3 | 74.89 (< .001) | Wachstumsklasse 1 vs. 4 | 87.64 (< .001) |
| Wachstumsklasse 2 vs. 4 | 7.19 (.007) | Wachstumsklasse 2 vs. 3 | 183.75 (<.001) |
| Wachstumsklasse 3 vs. 5 | 279.54 (.006) | Wachstumsklasse 2 vs. 5 | 278.33 (< .001) |
| Wachstumsklasse 4 vs. 5 | 422.20 (< .001) | Wachstumsklasse 3 vs. 5 | 8.95 (.003) |

*Anmerkungen.* Wachstumsklasse 1: Mathe hoch stabil/Französisch hoch Abnahme, Wachstumsklasse 2: Mathe hoch Abnahme/Französisch niedrig Abnahme, Wachstumsklasse 3: Mathe niedrig Abnahme/Französisch hoch stabil, Wachstumsklasse 4: Mathe mittel Abnahme/Französisch mittel Abnahme, Wachstumsklasse 5: Mathe niedrig Abnahme/Französisch niedrig Abnahme.

**Abb. A1** Dichteverteilungen mit Boxplots der Schüler*innen in den fünf Wachstumsklassen auf den Berufsorientierungen in Französisch und Mathematik


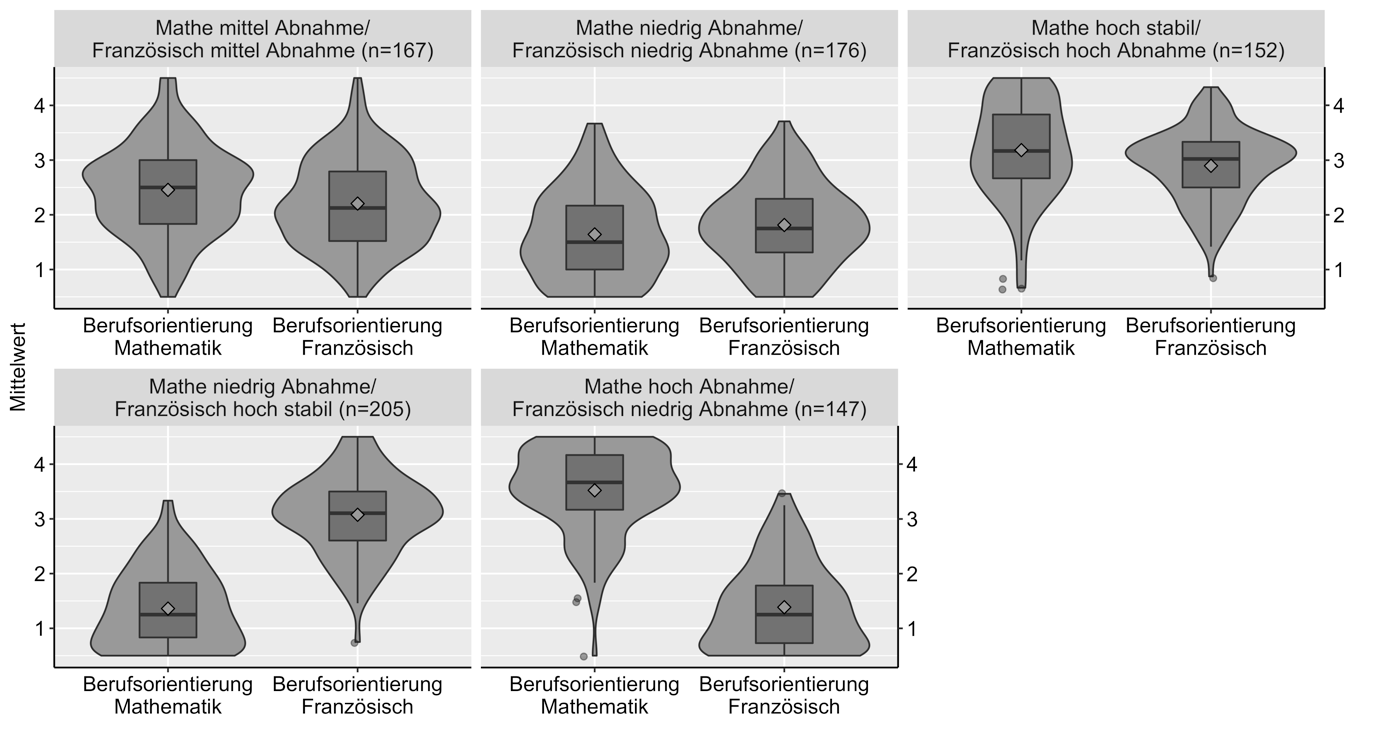

Supplement: Supplementary file 1 [file 11618_2022_1095_MOESM1_ESM.docx]
